# Integrated morbidity mapping of lymphatic filariasis and podoconiosis cases in 20 co-endemic districts of Ethiopia

**DOI:** 10.1371/journal.pntd.0006491

**Published:** 2018-07-02

**Authors:** Biruk Kebede, Sarah Martindale, Belete Mengistu, Biruck Kebede, Asrat Mengiste, Fikre H/Kiros, Abraham Tamiru, Gail Davey, Louise A. Kelly-Hope, Charles D. Mackenzie

**Affiliations:** 1 National Podoconiosis Action Network, Addis Ababa, Ethiopia; 2 Centre for Neglected Tropical Diseases, Department of Parasitology, Liverpool School of Tropical Medicine, Liverpool, United Kingdom; 3 Federal Ministry of Health, Addis Ababa, Ethiopia; 4 Wellcome Trust Centre for Global Health Research, Brighton & Sussex Medical School, Brighton, United Kingdom; 5 Michigan State University, East Lansing, Michigan, United States of America; RTI International, UNITED STATES

## Abstract

**Background:**

Lymphatic filariasis (LF) and podoconiosis are neglected tropical diseases (NTDs) that pose a significant physical, social and economic burden to endemic communities. Patients affected by the clinical conditions of LF (lymphoedema and hydrocoele) and podoconiosis (lymphoedema) need access to morbidity management and disability prevention (MMDP) services. Clear estimates of the number and location of these patients are essential to the efficient and equitable implementation of MMDP services for both diseases.

**Methodology/Principle findings:**

A community-based cross-sectional study was conducted in Ethiopia using the Health Extension Worker (HEW) network to identify all cases of lymphoedema and hydrocoele in 20 *woredas* (districts) co-endemic for LF and podoconiosis. A total of 612 trained HEWs and 40 supervisors from 20 districts identified 26,123 cases of clinical morbidity. Of these, 24,908 (95.3%) reported cases had leg lymphoedema only, 751 (2.9%) had hydrocoele, 387 (1.5%) had both leg lymphoedema and hydrocoele, and 77 (0.3%) cases had breast lymphoedema. Of those reporting leg lymphoedema, 89.3% reported bilateral lymphoedema. Older age groups were more likely to have a severe stage of disease, have bilateral lymphoedema and to have experienced an acute attack in the last six months.

**Conclusions/Significance:**

This study represents the first community-wide, integrated clinical case mapping of both LF and podoconiosis in Ethiopia. It highlights the high number of cases, particularly of leg lymphoedema that could be attributed to either of these diseases. This key clinical information will assist and guide the allocation of resources to where they are needed most.

## Author summary

Patients affected by the clinical conditions of lymphatic filariasis (lymphoedema and hydrocoele) and podoconiosis (lymphoedema) require access to a minimum package of care to prevent progression of the disease, and to improve their quality of life. Clear estimates of the number and location of these patients is essential for the delivery of this care. To address this, a community-based cross-sectional study was conducted in Ethiopia using the Health Extension Worker (HEW) network to identify all cases of lymphoedema and hydrocoele in 20 co-endemic *woredas* (districts). A total of 26,123 cases of clinical morbidity were identified. Of these, 24,908 (95.3%) had leg lymphoedema, of which 89.3% were bilateral. The results of this study will help assist the Neglected Tropical Disease (NTD) programme at the Federal Ministry of Health (FMOH) in Ethiopia to effectively and equitably plan the delivery of a basic package of care to those suffering from the clinical manifestations of both diseases.

## Introduction

Lymphatic filariasis (LF) and podoconiosis are neglected tropical diseases (NTDs) that affect the world’s poorest people and pose a significant economic burden to developing countries [1]. Responsible for 90% of cases worldwide, *Wuchereria bancrofti* is the causative agent of LF in Africa, and is transmitted through the bite of an infected mosquito. Infection with *W*. *bancrofti* can be either asymptomatic, or present as both acute and chronic clinical conditions. However, the main clinical conditions of LF disease that are recognised as significant public health priorities include acute dermatolymphangioadenitis (commonly known as acute attacks), lymphoedema and hydrocoele [2]. More than 36 million people worldwide are thought to be disfigured and incapacitated by this disease, leaving them vulnerable to an extremely complex range of physical, social and economic hardships [3, 4].

Podoconiosis, unlike LF, is not due to an infectious agent but rather is the result of specific inflammatory reactions to irritant mineral particles acquired from red clay soils derived from volcanic deposits [5–7]. Specifically, greater quantities of smectite, mica and quartz within the soil have been shown to have positive associations with podoconiosis prevalence [8]. It is found in highland tropical areas of Africa, Central America and north-west India where there is commonly a high seasonal rainfall [5, 6]. It is often termed “non-filarial” lymphoedema, and can be generally clinically distinguished from LF lymphoedema by its ascending progression of disease (rather than descending), and by being most commonly bilateral, as compared with LF which is most commonly descending and unilateral [7, 9]. As with LF, stigmatisation is a common and serious problem for those affected by podoconiosis, with patients experiencing suicidal thoughts, dissolution of marriage plans, often receiving insults and being excluded at social events [10]. Further, the economic consequences of the limb condition are profound, with productivity losses per patient amounting to 45% of working days per year, causing a monetary loss equivalent to US$ 63 per annum [11].

The World Health Assembly (WHA) adopted Resolution 50.29 in 1997, encouraging Member States to eliminate LF as a public health problem [12]. Soon after, the World Health Organisation (WHO) launched the Global Programme to Eliminate LF (GPELF) with a target date for achieving elimination by 2020. The two component strategies of GPELF are to a) interrupt transmission through mass drug administration (MDA), and b) to manage morbidity and prevent disability (MMDP) among those affected by the clinical manifestations of the disease. Although no global target has yet been set for the elimination of podoconiosis [7], strategies aimed at its control can be divided into primary, secondary and tertiary interventions. Primary intervention includes the use of footwear, regular foot hygiene and the application of floor coverings to reduce the contact between feet and the irritant soil. Secondary and tertiary interventions focus on the management of the lymphoedema-related morbidity and include wound care, exercise, elevation of the legs, treatment of acute attacks and providing psychosocial and socio-economic support to those affected [7].

As the minimum package of care for managing the lymphoedema seen in LF and podoconiosis patients to manage morbidity is very similar, it is practical that MMDP should be integrated to help improve cost-effectiveness and extend the reach of the programme [2]. This is particularly important in Ethiopia where there is a high burden of both diseases, with 29 of the 70 LF-endemic districts considered to be co-endemic [13, 14]. However, before MMDP activities for both diseases can be implemented, better patient estimates and an understanding of the co-distributions of both diseases at district level are vital. To assist the NTD programme at the Federal Ministry of Health (FMOH) to effectively and equitably plan the delivery of a basic package of care to those suffering from clinical manifestations of both diseases, we have examined the clinical burden of lymphoedema and hydrocoele in 20 co-endemic *woredas* (districts) of Ethiopia.

## Methods

### Ethics statement

Ethical approval for this study was obtained from the Research Ethics Committee at the Liverpool School of Tropical Medicine, UK (Research Protocol 12.22), and the Amhara and SNNP Regional Health Bureaus in Ethiopia. Informed consent was obtained from all household heads and patients involved in the study. All adult participants provided informed consent, no children participated in the study. Participants who could read and write provided written consent. Those who could not read and write, had the consent information read to them and oral consent was recorded. The methods of collecting oral and written consent was approved by the Regional Health Bureau ethical committees.

### Study site characteristics

**Fig 1:**
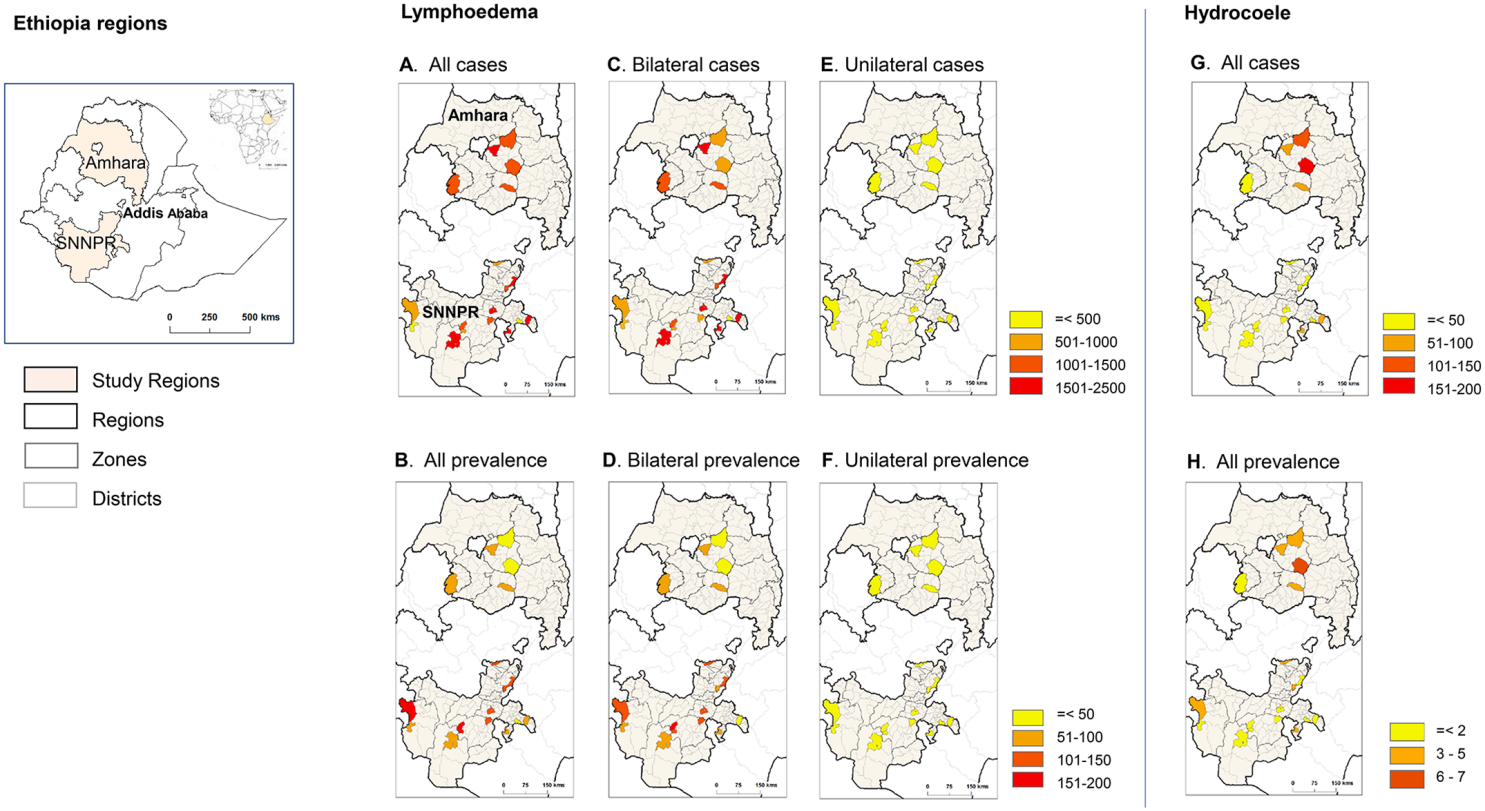
Study regions in Ethiopia, and the number and prevalence per 10,000 of lymphoedema and hydrocoele cases by district. A. Lymphoedema–all cases. B. Lymphoedema–all prevalence. C. Lymphoedema–bilateral cases. D. Lymphoedema–bilateral prevalence. E. Lymphoedema–unilateral cases. F. Lymphoedema–unilateral prevalence. G. Hydrocoele–all cases. H. Hydrocoele–all prevalence. Note. The names of each district and their endemicity classification are shown in reference S1 Fig.

The study was conducted in the Southern Nations, Nationalities, and Peoples’ region (SNNPR) and Amhara region of Ethiopia (Fig 1) between May and August 2015. In total 20 districts were selected; 14 districts from eight zones in the SNNPR, and six districts from three zones in Amhara region, which had a total population of 3,075,318 (50.2% male) [15, 16].

At the time of the study, the 20 districts were considered to be co-endemic for LF and podoconiosis, with LF prevalence based on mapping conducted using immunochromatographic card tests (ICT; Alere, Scarborough, ME, USA) to detect the presence of circulating *W*. *bancrofti* antigen as per the WHO protocol [17–19]. However, due to borderline endemicity in districts, which had exactly the 1% prevalence threshold for endemicity, a subsequent ‘re-mapping’ validation was undertaken in nine of these 20 districts before the scale-up of MDA [14]. These nine districts were: Bensa, Saula Town (Sawla), Kebena, South Ari (Debub Ari), Guanga (Guangua), Ebinat, Fogera, Simada (Semada) and E/Enawga (Inarj-Inawuga). This ‘re-mapping’ resulted in seven of these districts being reclassified as non-endemic, while two (South Ari and Simada), have remained endemic. The name and distribution of the non-endemic and endemic districts are shown in S1 Fig [14]. With regards to the clinical burden of disease, prior to the present study, no comprehensive information was available on the number of clinical cases in these districts, and no available service provision for MMDP of lymphoedema and hydrocoele. Before 2015, LF MDA had been initiated in only two of the 20 districts, Bero district and Guraferda district of the Bench Maji zone. This MDA was integrated with the onchocerciasis control programme.

### Study design and tools

The community-based cross-sectional study was conducted using the Health Extension Worker (HEW) network to identify cases. HEWs are predominately female, and are recruited from the communities they serve to expand access to basic health promotion, disease prevention and selected curative health services [20]. The HEWs searched for patients affected by LF and podoconiosis in their community catchment areas, with two HEWs covering a population of approximately 5,000 people [20]. Generally, two HEWs are assigned to cover one *kebele*. The HEWs used a patient information collection form (translated into Amharic) to record personal details including general demographic information (sex, age, occupation, rural/urban residence, education level), and the condition of the patient (leg lymphoedema only, breast lymphoedema, hydrocoele or ‘both’ leg lymphoedema and hydrocoele), and whether leg lymphoedema was unilateral or bilateral. Patients were also asked whether they had suffered an acute attack in the past six months because of their swelling or lymphoedema.

The severity of leg lymphoedema was staged as mild, moderate or severe using WHO recommended guidelines [21]. Patients with mild lymphoedema were categorised as those with a slight, soft swelling; whilst moderate lymphoedema was an enlarged swelling with shallow folds; and severe lymphoedema being greatly enlarged, with deep folds, and skin changes including mossy lesions and nodules. HEWs were given an image of the three stages of lymphoedema to assist them in recording the staging. Due to the sensitivity of the condition, HEWs were not asked to stage or confirm the presence of the hydrocoele, but just asked to record the men reporting scrotal swelling. HEWs were not asked to differentiate between the causes of lymphoedema (podoconiosis or lymphatic filariasis) as currently podoconiosis is a diagnosis of clinical exclusion based on history, physical examination and certain disease-specific tests to exclude common differential diagnoses [18]. The use of such a complex clinical algorithm, previously used in epidemiological mapping of LF and podoconiosis in Ethiopia [18] was considered inappropriate for community-based health worker patient searching.

### Training

In each district, all HEWs and two supervisors attended a one-day training session. The training focused on: a) how to identify lymphoedema and hydrocoele; b) how to classify lymphoedema into the three stages; and c) how to record the patient information on the collection form. In addition, all attendees were trained on morbidity management (basic care package) for LF and podoconiosis to enable HEWs to give advice at the time of identification regarding basic lymphoedema management, or to refer any suspected hydrocoele cases to their nearest health facility for validation and further referral for surgery.

### Data collection and analysis

Following the training, each HEW returned to her respective catchment area to identify all cases of lymphoedema and hydrocoele through house-to-house patient searching; with the two supervisors overseeing the data collection and acting as a point of contact if any problems were encountered. The two HEWs assigned to each *kebele* as per the standard government health system, were assigned to identify patients in the households they usually serve. For each *kebele*, the HEWs used the list of households available in each *kebele* to prevent overlap between the HEWs and to ensure all households were covered. During the data collection, each HEW wrote the name of each household head on the data collection form, and this was cross-checked against the list of households by the trained supervisors. To ensure no households were missed, HEWs sought support from the Health Development Army (volunteer community health promoters) and the *kebele* administrative team to inform each specific household of the date and time the HEW would visit the household for the patient searching. The supervisors also visited a selection of the households to ensure they had been visited by the HEWs.

The assessment was completed within ten days in most of the districts, with the data collection forms collated soon afterwards. All data was entered into Microsoft Excel Version 12.3.6 (Microsoft Corp., Redmond, VA, USA) and analysed using IBM SPSS Statistics 32 (IBM Corp., Armonk, NY, USA). Prevalence estimates (per total 10,000 population) were calculated using the 2015 population estimates as the denominator, which were calculated from the 2007 census [15] using the annual growth rates [16].

Statistical analysis between regions and variables including condition, severity of lymphoedema, acute attacks, sex and age were examined using Pearson’s chi square with p value <0.05, and/or odds ratios (OR) with 95% confidence intervals (95%CIs) to determine levels of significance. Maps highlighting the study regions, and the number and prevalence of cases with lymphoedema and hydrocoele per 10,000 of the total population were produced using geographical information system (GIS) software ArcGIS 10 (ESRI., Redlands, CA, USA). Further overall, lymphoedema and hydrocoele prevalence rates were compared between the newly classified non-endemic districts and the endemic districts. In addition, to account for the different geographical sizes of the districts and to better understand the density of conditions, the number of cases per square kilometre (km^2^) was calculated and presented in a supplementary file (S1 Table).

## Results

### Summary of reported cases

A total of 612 HEWs and 40 supervisors from 20 districts were trained to collect and report case data. Table 1 presents the number of cases reported per clinical condition for each region, zone and district. A total of 26,123 cases (48% male; mean age 43.6 years) were reported. For the SNNPR, the total was 17,285 cases (44.6% male; mean age 51.2 years) and for Amhara region was 8,838 cases (54.8% male; mean age 43.8 years). Overall, the majority of cases were either subsistence farmers (n = 17,243; 66%) or housewives (n = 5,557; 21.3%), had permanently lived in the rural setting (n = 24,198; 92.6%), had not attended school and were illiterate (n = 21,631; 82.8%)

**Table 1:** Reported number and prevalence (per 10,000 of the total population) of clinical cases.

Region	Zone	#	District	Total population	Leg lymphoedema only		Hydrocoele only		Both conditions**		Breast lymphoedema only		Total	
					N	Prevalence	N	Prevalence*	N	Prevalence	N	Prevalence	N	Prevalence
SNNPR	Siliti	1	Lanfuro	142,581	1,781	124.9	20	1.4	3	0.2	1	0.1	1,805	126.6
		2	Sankura	104,051	1,130	108.6	23	2.2	17	1.6	0	0.0	1,170	112.4
	Sidama	3	Hawela Tula	152,844	405	26.5	4	0.3	1	0.1	0	0.0	410	26.8
		4	Bensa ǂ	307,878	1,882	61.1	54	1.8	29	0.9	2	0.1	1,967	63.9
	Gedeo	5	Yirga Chefe	239,763	1,699	70.9	57	2.4	13	0.5	0	0.0	1,769	73.8
	Wollaita	6	Sodo zuria	199,775	2,564	128.3	12	0.6	5	0.3	0	0.0	2,581	129.2
	GamoGofa	7	Boreda	83,451	1,078	129.2	14	1.7	6	0.7	25	3.0	1,123	134.6
		8	Gezegofa	84,298	1,343	159.3	11	1.3	32	3.8	2	0.2	1,388	164.7
		9	Oyida	40,903	624	152.6	7	1.7	18	4.4	0	0.0	649	158.7
		10	Saula Town ǂ	27,879	247	88.6	1	0.4	0	0.0	0	0.0	248	89.0
	Gurage	11	Kebena ǂ	64,318	892	138.7	29	4.5	10	1.6	0	0.0	931	144.7
	Bench Maji	12	Bero	15,050	105	69.8	0	0.0	0	0.0	0	0.0	105	69.8
		13	Guraferda	43,311	663	153.1	12	2.8	4	0.9	0	0.0	679	156.8
	South Omo	14	South Ari	258,552	2,277	88.1	41	1.6	101	3.9	42	1.6	2,461	95.2
**SNNP regional total**				**1,764,654**	**16,690**	**94.6**	**284**	**1.6**	**239**	**1.4**	**72**	**0.4**	**17,285**	**98.0**
Amhara	Awi	15	Zigem	82,174	1,203	146.4	34	4.1	23	2.8	2	0.2	1,262	153.6
		16	Guanga ǂ	191,738	1,487	77.6	38	2.0	54	2.8	3	0.2	1,582	82.5
	South Gondor	17	Ebinat ǂ	270,365	1,080	39.9	112	4.1	6	0.2	0	0.0	1,198	44.3
		18	Fogera ǂ	280,522	1,932	68.9	72	2.6	31	1.1	0	0.0	2,035	72.5
		19	Simada	280,304	1,033	36.9	157	5.6	10	0.4	0	0.0	1,200	42.8
	East Gojam	20	E/Enawga ǂ	205,560	1,483	72.1	53	2.6	24	1.2	0	0.0	1,560	75.9
**Amhara regional total**				**1,310,663**	**8,218**	**62.7**	**467**	**3.6**	**148**	**1.1**	**5**	**0.04**	**8,838**	**67.4**
**Overall total**				**3,075,318**	**24,908**	**81.0**	**751**	**2.4**	**387**	**1.3**	**77**	**0.3**	**26,123**	**84.9**

* Hydrocoele prevalence based on total population

** Both refers to patients identified as having both hydrocoele and lymphoedema conditions

^ǂ^ Remapping indicated that these districts were non-endemic [14]

In terms of clinical conditions, the total number of cases reported comprised of 24,908 (95.3%) leg lymphoedema, 751 (2.9%) hydrocoele, 387 (1.5%) both leg lymphoedema and hydrocoele, and 77 (0.3%) breast lymphoedema. No individual patient was reported as having both a breast lymphoedema and a leg lymphoedema, and from this point onwards, the term ‘both conditions’ refers to a patient having both a leg lymphoedema and a hydrocoele. The maps shown in Fig 1A–1H highlight the case and prevalence distributions across the study area. The districts with the highest number of cases were Sodo Zuria district, Wollaita zone (n = 2,581), and South Ari district, South Omo zone (n = 2,461) of the SNNPR (Table 1). The districts with the lowest number of cases were Bero district, Bench Maji zone (n = 105), and Saula Town, GamoGofa zone (n = 248) of the SNNPR. The distribution of all cases by district is shown in Fig 1A.

Overall, the morbidity prevalence rate was 84.9 per 10,000 total population (Table 1). The SNNPR rate of 98.0 per 10,000 was significantly higher than the Amhara region rate of 67.4 per 10,000 (OR 1.46; 95%CIs 1.42–1.50; P< 0.0001). The districts with the highest prevalence rates were Gezegofa district (164.7 per 10,000), and Oyida district (158.7 per 10,000) in the GamoGofa zone of the SNNPR, while the district with the lowest was Hawela Tula district, Sidama zone (26.8 per 10,000) of the SNNPR. The distribution of all cases is shown in Fig 1B. When comparing by endemicity status, the overall prevalence rate in the non-endemic districts of 70.6 per 10,000 was significantly lower than the endemic districts (96.1 per 10,000) (OR 0.73; CIs 0.71–0.75; P<0.0001).

The density of cases overall was 17.53 cases per km^2^ (see S1 Table). The SNNPR (23.60 cases per km^2^) had a higher density of cases than the Amhara region (11.66 cases per km^2^). The highest district level density of cases were in the urban areas, Saula Town, GamoGofa zone (634.85 per km^2^) of the SNNPR, and Zigem, Awi zone (467.11 per km^2^) of the Amhara region. The lowest density of cases were in Bero district (2.18 cases per km^2^), and Guraferda district (3.35 cases per km^2^) of the Bench Maji zone of the SNNPR.

### Lymphoedema cases

#### Summary

Overall, leg lymphoedema (only) accounted for most of the cases reported and was found in similar patterns to the total number of cases and prevalence rates described above and shown in Table 1. The districts with the highest number of cases were Sodo zuria district, Wollaita zone (n = 2,564), and the lowest was Bero district, Bench Maji zone (n = 105) of the SNNPR. Similarly, the overall lymphoedema prevalence rate was 81.0 per 10,000 total population. The SNNPR rate of 94.6 per 10,000 was significantly higher than the Amhara region rate of 62.7 per 10,000 (OR 1.51; 95%CIs 1.47–1.55; P< 0.0001). The district with the highest prevalence rate was Gezegofa district, GamoGofa zone (159.3 per 10,000), and the lowest was Hawela Tula district, Sidama zone (26.5 per 10,000) of the SNNPR. When comparing by endemicity status, the lymphoedema prevalence rate in the non-endemic districts (66.8 per 10,000) was significantly lower than the endemic districts (92.1 per 10,000) (OR 0.73; CIs 0.71–0.74; P<0.0001). In addition, similar trends in the density of lymphoedema cases was found as the overall total as shown in S1 Table.

**Fig 2:**
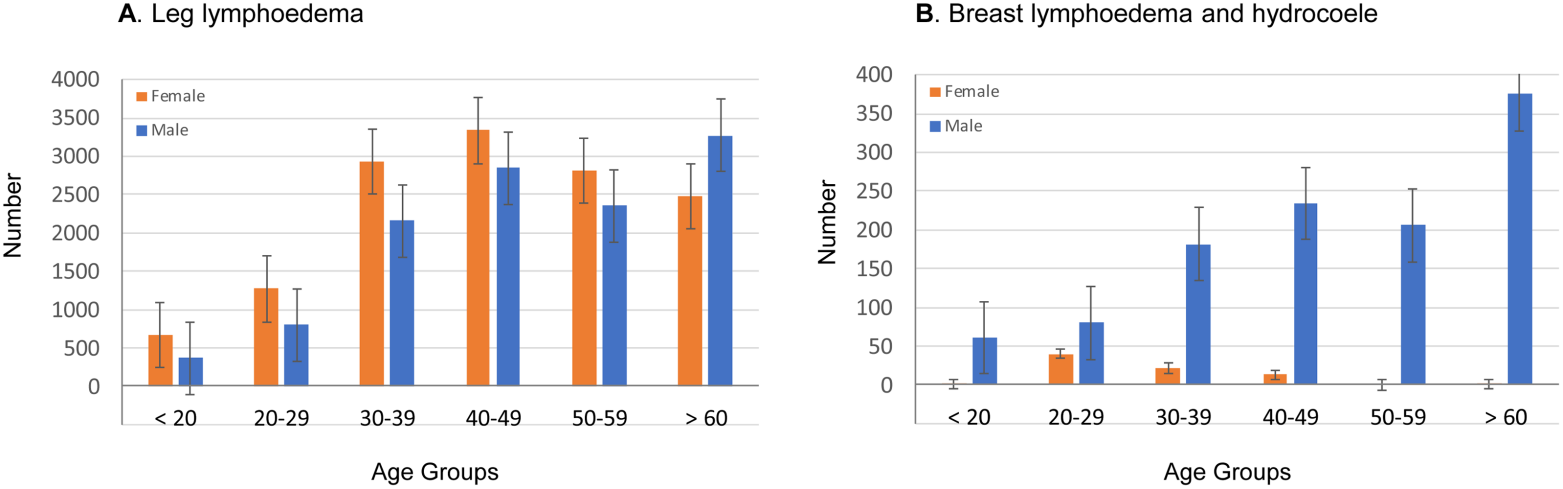
Number of leg lymphoedema, breast lymphoedema and hydrocoele cases by sex and age. A. Leg lymphoedema. B. Breast lymphoedema and hydrocele.

Overall, more females (54.2%; n = 13,495) reported leg lymphoedema (only) than males. The majority of cases were aged above 30 years of age, with similar patterns for both males and females. The distribution of leg lymphoedema by age and sex is shown in Fig 2A. A small number of male cases reported having both a leg lymphoedema and a hydrocoele (n = 387) in 18 of the 20 districts. A smaller number of females reported having breast lymphoedema (n = 77) in 7 of the 20 districts, with most recorded in South Ari district, South Omo zone (n = 42) and Boreda district, GamaGofa zone (n = 25) of the SNNPR (Table 1). The distribution of breast lymphoedema and hydrocoele cases by age and sex is shown in Fig 2B.

#### Number of limbs affected by leg lymphoedema

In total, 25,295 cases reported leg lymphoedema, including those with leg lymphoedema (only), and those with both leg lymphoedema and hydrocoele. Of these, 89.3% (n = 22,597) reported they had bilateral leg lymphoedema with similar percentages found in both the SNNPR (89.9%) and Amhara region (88.1%). More variation at district level was reported with the percentage of bilateral leg lymphoedema cases ranging from 80.2% to 96.1%. The number and distribution of unilateral and bilateral cases by district are summarised in Table 2 and shown in Fig 1A, 1C and 1E.

**Table 2:** Reported number, prevalence (per 10,000 of the total population) and ratio of unilateral and bilateral cases of leg lymphoedema.

Region	Zone	District	Total population	Leg lymphoedema*					Bilateral % of total	Unilateral: bilateral ratio (n)
				Unilateral		Bilateral		Total		
				N	Prevalence	N	Prevalence			
SNNPR	Siliti	Lanfuro	142,581	186	13.0	1,598	112.1	1,784	89.6%	1: 9
		Sankura	104,051	116	11.1	1,031	99.1	1,147	89.9%	1: 9
	Sidama	Hawela Tula	152,844	16	1.0	390	25.5	406	96.1%	1: 24
		Bensa ǂ	307,878	379	12.3	1,532	49.8	1,911	80.2%	1: 4
	Gedeo	Yirga Chefe	239,763	77	3.2	1,635	68.2	1,712	95.5%	1: 21
	Wollaita	Sodo zuria	199,775	245	12.3	2,324	116.3	2,569	90.5%	1: 9
	GamoGofa	Boreda	83,451	123	14.7	961	115.2	1,084	88.7%	1: 8
		Gezegofa	84,298	93	11.0	1,282	152.1	1,375	93.2%	1: 14
		Oyida	40,903	54	13.2	588	143.8	642	91.6%	1: 11
		Saula Town ǂ	27,879	13	4.7	234	83.9	247	94.7%	1: 18
	Gurage	Kebena ǂ	64,318	138	21.5	764	118.8	902	84.7%	1: 6
	Bench Maji	Bero	15,050	9	6.0	96	63.8	105	91.4%	1: 11
		Guraferda	43,311	60	13.9	607	140.1	667	91.0%	1: 10
	South Omo	South Ari	258,552	193	7.5	2,185	84.5	2,378	91.9%	1: 11
**SNNP regional total**			**1,764,654**	**1,702**	**9.6**	**15,227**	**86.3**	**16,929**	**89.9%**	**1: 9**
Amhara	Awi	Zigem	82,174	113	13.8	1,113	135.4	1,226	90.8%	1: 10
		Guanga ǂ	191,738	81	4.2	1,460	76.1	1,541	94.7%	1: 18
	South Gondor	Ebinat ǂ	270,365	190	7.0	896	33.1	1,086	82.5%	1: 5
		Fogera ǂ	280,522	212	7.6	1,751	62.4	1,963	89.2%	1: 8
		Simada	280,304	184	6.6	859	30.6	1,043	82.4%	1: 5
	East Gojam	E/Enawga ǂ	205,560	216	10.5	1,291	62.8	1,507	85.7%	1: 6
**Amhara regional total**			**1,310,663**	**996**	**7.6**	**7,370**	**56.2**	**8,366**	**88.1%**	**1: 7**
**Overall total**			**3,075,318**	**2,698**	**8.8**	**22,597**	**73.5**	**25,295**	**89.3**%	**1: 8**

*Leg lymphoedema including those with leg lymphoedema only and both conditions (lymphoedema and hydrocoele)

^ǂ^ Remapping indicated that these districts were non-endemic [14]

The overall ratio of unilateral to bilateral leg lymphoedema cases was 1:8, which was similar between the SNNPR (1:9) and Amhara region (1:7). Similarly, there was more variation at district level with ratios ranging from 1:4 to 1:24, with the highest reported in Hawela Tula district, Sidama zone (1:24) and Yirga Chefe district, Gedeo zone (1:21) and the lowest in Bensa district, Sidama zone of the SNNPR (1:4).

Similarly, the overall prevalence of unilateral (8.8 per 10,000) and bilateral (73.5 per 10,000) lymphoedema cases differed, with similar large differences between unilateral and bilateral leg lymphoedema within each region. When comparing regions, a significantly higher prevalence of unilateral lymphoedema was reported in the SNNPR (9.6 per 10,000) compared with Amhara region (7.6 per 10,000) (OR 1.27; CIs 1.17–1.37; P<0.0001). Similarly, a significantly higher prevalence of bilateral lymphoedema was reported in the SNNPR (86.3 per 10,000) compared with Amhara region (56.2 per 10,000) (OR 1.54; CIs 1.50–1.58; P<0.0001). The overall prevalence and distribution of all, unilateral and bilateral cases are summarised in Table 2 and shown in Fig 1B, 1D and 1F.

#### Severity of leg lymphoedema

The number of cases (n = 25,295) reporting mild, moderate or severe leg lymphoedema (including those with leg lymphoedema only and both leg lymphoedema and hydrocoele) varied significantly (chi square <0.05). Over half of the cases were recorded as mild (57.8%; n = 14,492), approximately one third as moderate (33.8%; n = 8,538) and 9% as severe (n = 2,265). The proportion of male and female cases for each stage was similar, with just over half of mild, moderate and severe cases being female; 54.1%, 52.6% and 51.6%, respectively.

In terms of age, significant differences were found in the mean age and the different stages of leg lymphoedema, with an increasing trend in the severity of the condition by age group. The mean age of cases reporting mild, moderate and severe leg lymphoedema was 45.1 years (95% CI 44.8–45.3), 47.6 years (95% CI 47.3–47.9) and 49.0 years (95% CI 48.3–49.6) respectively.

### Hydrocoele cases

In all 20 districts, the total number and prevalence of men reporting hydrocoele was low with only 751 cases reported overall and a prevalence of 2.4 per 10,000 population (Table 1). The SNNPR rates of 1.6 per 10,000 was significantly lower than the Amhara region rates of 3.6 per 10,000 (OR 0.45; 95%CIs 0.39–0.52; p< 0.0001). The two districts with the highest number of cases and prevalence rates were Simada district (n = 157; 5.6 per 10,000), and Ebinat district (n = 112; 4.1 per 10,000), South Gondor zone in Amhara region. Of note, Ebinat district was recently re-classified as ‘non-endemic’ for LF [14]. Bero district, Bench Maji zone in the SNNPR reported no cases. All reported cases of hydrocoele were male with the majority aged 41 to 60 years (41.9%; n = 315) The number of cases reported increased with age as highlighted in Fig 2B. When comparing by endemicity status, the hydrocoele prevalence rate in the non-endemic districts of 2.7 per 10,000 was significantly higher than the endemic districts of 2.3 per 10,000 (OR 1.2; CIs 1.0–1.4; P = 0.029).

The density of hydrocoele cases overall was very low at 0.5 cases per km^2^ (see S1 Table). The SNNPR (0.39 cases per km^2^) had a lower density of cases than the Amhara region (0.62 cases per km^2^). The highest district level density of cases were in the urban areas, Saula Town, GamoGofa zone (2.56 cases per km^2^) of the SNNPR, and Zigem, Awi zone (12.58 cases per km^2^) of the Amhara region. The lowest density of cases was in Guraferda district (0.06 cases per km^2^), Bench Maji zone of the SNNPR.

### Acute attacks

Of all the 26,123 cases identified, a total of 1,931 cases did not record any reliable information on acute attacks in the last six months, and therefore were excluded from this analysis (Table 3). Of the 24,192 cases with information recorded, approximately two-thirds (64.1%; n = 15,517/24,192) reported that they had at least one acute attack in the last six months due to their swelling/lymphoedema. Similar proportions were recorded for those with leg lymphoedema (64.5%; n = 14,935/23,165), and/or both conditions (65.3%; n = 211/323). Men with hydrocoele (54.5%; n = 343/629) and women with breast lymphoedema (37.3%; n = 28/75) reported fewer acute attacks.

**Table 3:** Reported acute attacks for all conditions by different age groups and by sex.

Overall			Differences by sex					
Age group	Total cases	No. Positive (%)	Sex	Subtotal (n)	Total positive	Positive %	Age–sex OR (95% CI)	P value
<20	974	549 (56.4%)	M	368	185	50.3%	1.49 (1.15–1.93)	0.0029*
			F	606	364	60.1%		
20–29	1,969	1,155 (58.7%)	M	756	416	55.0%	1.27 (1.06–1.53)	0.0098*
			F	1,213	739	60.9%		
30–39	4,803	2,911 (60.6%)	M	2,068	1,199	58.0%	1.21 (1.07–1.36)	0.0012*
			F	2,735	1,712	62.6%		
40–49	5,865	3,723 (63.5%)	M	2,732	1,668	61.1%	1.21 (1.09–1.35)	0.0003*
			F	3,133	2,055	65.6%		
50–59	4,921	3,220 (65.4%)	M	2,318	1,459	62.9%	1.23 (1.09–1.39)	0.0005*
			F	2,603	1,761	67.7%		
>60	5,660	3,959 (69.9%)	M	3,308	2,285	69.1%	1.11 (0.98–1.24)	0.0898
			F	2,352	1,674	71.2%		
**Total**	**24,192**	**15,517 (64.1%)**	**M**	**11,550**	**7,212**	**62.4%**	**1.13 (1.07–1.19)**	<0.0001*
			**F**	**12,642**	**8,305**	**65.7%**		

A total of 1,931 participants did not record information on acute attacks and therefore were excluded from the analysis. In the table, positive refers to patients who reported experiencing at least one acute attack in the last six months.

* Statistically significant at <0.05

**Fig 3:**
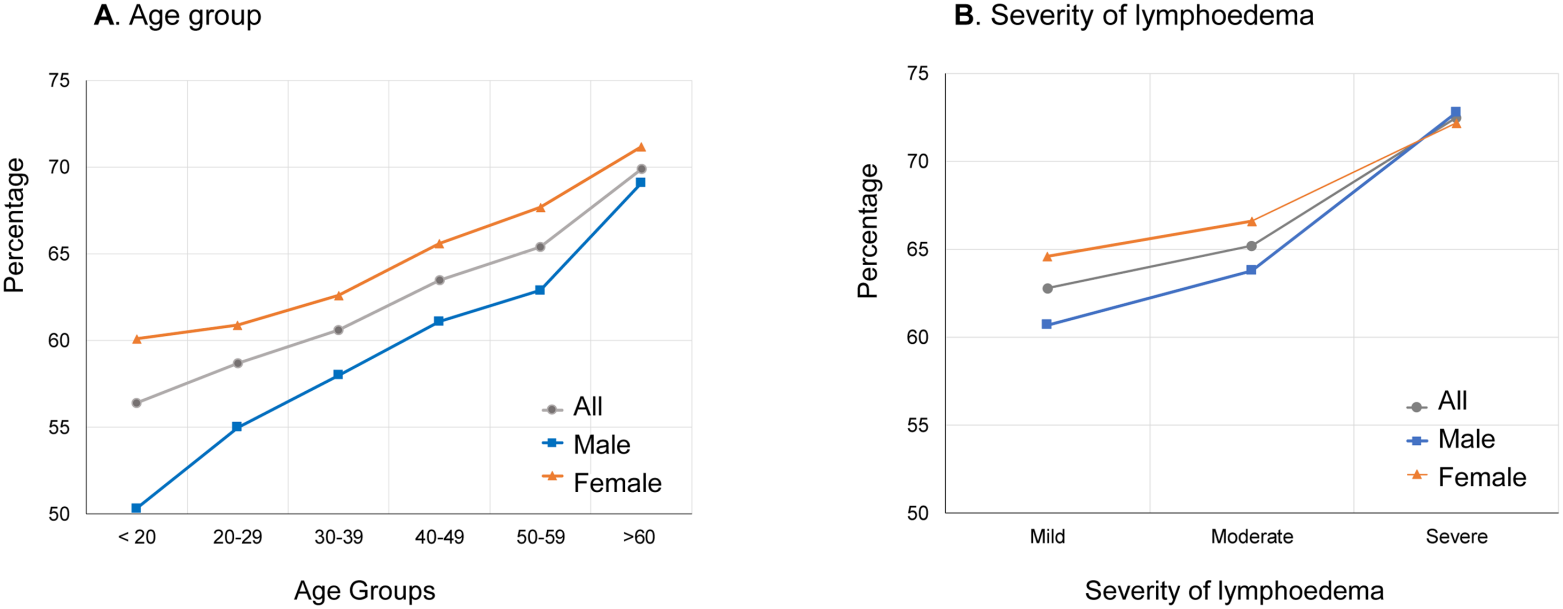
Proportion of cases experiencing acute attacks by age group and severity of lymphoedema stratified by sex. A. Age group. B. Severity of lymphoedema.

Overall, a higher percentage of females reported they had an acute attack in the last six months (65.7%; n = 8,305/12,642) than males (62.4%; n = 7,212/11,550), this was a significant difference (OR 1.13; 95%CIs 1.07–1.19) (Table 3). Overall, the percentage of acute attacks increased with age, which was similar for both males and females (Fig 3A). The lowest percentage of acute attacks reported were in the <20 year age group (56.4%) and the highest in the older >60 year age group (69.9%). A significantly higher percentage of females reported acute attacks than males across all age groups; an exception to this was the >60 year age group as shown in Table 3.

In terms of severity of leg lymphoedema, a total of 23,488 cases had information recorded (Table 4). Overall, the percentage of acute attacks reported increased with the severity of the condition; mild (62.8%; n = 8,529), moderate (65.2%; n = 5,111) and severe (72.5%; n = 1,506) as shown in Fig 3B. A significantly higher percentage of females reported acute attacks with mild (OR 1.17; 95%CIs 1.10–1.26) and moderate (OR 1.12; 95%CIs 1.02–1.23) conditions than males, however no difference by sex was found with severe conditions.

**Table 4:** Reported leg lymphoedema severity and acute attacks of reported cases.

Severity	Overall		Differences by sex					
	Total cases	No. positive (%)	Sex	Subtotal (n)	Total positive	Positive %	OR (95% CI)	P value
Mild	13,577	8,529 (62.8%)	M	6223	3780	60.7%	1.17 (1.10–1.26)	<0.0001*
			F	7354	4749	64.6%		
Moderate	7,833	5,111 (65.2%)	M	3698	2361	63.8%	1.12 (1.02–1.23)	0.0136*
			F	4135	2750	66.5%		
Severe	2,078	1,506 (72.5%)	M	1000	728	72.8%	0.97 (0.80–1.17)	0.748
			F	1078	778	72.2%		
**Total**	**23,488**	**15,146 (64.5%)**	**M**	**10921**	**6869**	**62.9%**	**1.14 (1.08–1.20)**	<0.0001*
			**F**	**12567**	**8277**	**65.9%**		

Leg lymphoedema included those cases reporting leg lymphoedema only and both conditions. In total 1,807 cases of 25,295 total number were excluded from the analysis as they did not record information on acute attacks. In the table, positive refers to patients who reported experiencing at least one acute attack in the last six months.

* Statistically significant at <0.05

## Discussion

This study represents the first community-wide integrated clinical case survey of both LF and podoconiosis in Ethiopia. In the 2007 epidemiological mapping of LF in 112 districts of Western Ethiopia, only advanced cases of LF-related lymphoedema and hydrocoele were reported from selected villages within the surveyed districts [17]. Later, during the integrated endemicity mapping in 658 districts in 2013, clinical cases of LF and podoconiosis had only been reported from two *kebeles* per district using a two-stage cluster purposive sampling strategy [18, 19]. Other studies assessing the clinical burden of either LF or podoconiosis individually have generally only been conducted at a smaller geographical scale, and only in districts known to be endemic for the specific disease [22–27].

The present survey results highlight a greater burden of lymphoedema cases compared to that of hydrocoele, with over 33 times as many leg lymphoedema cases reported than hydrocoele. These results differ to other studies on clinical case estimates in African LF-endemic countries including Malawi and Tanzania where the number of hydrocoele cases identified was almost double that of lymphoedema cases identified [28, 29].This is likely to be related to the presence of podoconiosis in Ethiopia. Here we highlight that the vast majority of lymphoedema cases were bilateral; a manifestation more typically seen with non-filarial lymphoedema [9]. Although these results suggest that a clear majority of lymphoedema cases identified are likely to be caused by podoconiosis rather than filariasis, this present study did not distinguish between the causes of lymphoedema. These results are supported by previous findings by Deribe *et al* [30], which highlights the huge burden of podoconiosis in the SNNP and Amhara regions of Ethiopia, particularly in the central highland area where distinct environmental and climatic factors are suitable for podoconiosis occurrence. LF tests including circulating filarial antigen testing, filarial antibody examination and parasitological examination have been used to exclude an LF diagnosis in other studies in LF and podoconiosis co-endemic areas [31], but for the purposes of understanding the burden of lymphoedema to establish MMDP interventions in this study, a detailed understanding of aetiology was not required as the same MMDP interventions are required for both filarial and non-filarial lymphoedema.

Regardless of aetiology, the high number of leg lymphoedema cases highlights the pressing need to deliver a basic package of care to those suffering from these disabling conditions, especially in areas with a high prevalence and/or high density of conditions where patients may be more readily found and the distribution of care easier. The delivery of a low-cost lymphoedema management programme based on limb washing and topical medication for infection has been shown to reduce the number of debilitating acute attacks and to increase the economic productivity of patients [32]. An integrated MMDP programme will help the majority of lymphoedema cases in these districts as most were identified as having a mild severity and are likely to respond well to such treatment [33]. Such an MMDP programme should be fully integrated into the existing health system structure to ensure sustainability and to help achieve universal health coverage. It should also promote early detection of mild lymphoedema cases, which may be underreported by HEWs, to prevent progression to a more severe stage of lymphoedema.

A verification exercise to confirm reported cases of lymphoedema and hydrocoele by a clinician has been used in other studies to validate the reporting method used for patient searching [28, 29] but due to financial and time constraints, was not included in this study. Such verification exercises have evidenced that community health workers are exceptionally well-placed to participate in quantifying LF morbidity burden, and other NTDs with observable symptoms [29], such as podoconiosis. As other countries begin to develop their own methods for obtaining patient estimates to achieve the second component of the GPELF strategy, this study has highlighted that integrated patient searching using community health workers is an effective strategy. By including two diseases, this method is cost-effective and empowers local health workers to contribute to local, national and global elimination of disease.

In this study, those with more severe disease were shown to be more likely to have experienced an acute attack in the past six months. This is not a surprising result as the presence of moisture, which commonly accumulates in swollen folds of skin, or the web spaces of toes during lymphoedema promotes fungal infections, leading to an acute attack [34–36]. Also, older age groups were shown to have a more severe stage of disease, were more likely to have bilateral lymphoedema, and were more likely to have experienced an acute attack in the past six months. As lymphoedema is a chronic, progressive condition, this is to be anticipated and highlights the important need to implement an accessible package of care to all lymphoedema patients to prevent the progression of their condition to a more disabling and debilitating severe stage.

The low number of hydrocoele cases identified in this study further suggests that LF is of low prevalence in these regions of Ethiopia, and that with well-targeted morbidity strategies, GPELF targets could be reached. However, it is important to consider that as hydrocoele is a highly stigmatised condition [37], the numbers reported in this study could be an underestimation. As HEWs are predominately female and likely to be from the same community as the patient [20], some patients may not have disclosed their condition to their HEW. For those hydrocoele cases identified in this study and those that may remain hidden, it is important to identify equitable ways to refer and facilitate access to safe hydrocoele surgery to repair their condition. In an effort to support this, international partners have worked together with the Federal Ministry of Health (FMOH) in Ethiopia to develop a hydrocoele surgical handbook and trained regional surgeons across Ethiopia on surgical best practise [38]. Such surgery has been shown to positively impact patients’ lives, improving their physical and socio-economic output [39] and to be highly cost-effective [40]. Facilitating access to surgery in the districts now classified as ‘non-endemic’ will be particularly important as patients could miss out on treatment if resources are not prioritised to these districts, especially as the prevalence rates were higher than the endemic districts. However, it will still remain important to provide services for lymphoedema patients in these regions also, whether they are caused by LF or podoconiosis.

In order to achieve the second pillar of the GPELF plan, services to alleviate the suffering of clinical disease needs to be available within primary health care systems in all areas of known patients [41]. The results of this study highlight the profound burden of clinical disease, particularly lymphoedema. Since 2015, funds have been mobilised by the Centre for Neglected Tropical Diseases at the Liverpool School of Tropical Medicine (LSTM) with funding from the Department for International Development (UKAID) to provide lymphoedema and hydrocoele patients across all 20 districts with access to care. The results of this study have therefore helped to improve the management of both LF and podoconiosis morbidity, and will assist the NTD programme in Ethiopia to achieve elimination goals.

## Supporting information

S1 Checklist STROBE checklist. (DOC)

S1 Fig Map highlighting district names and endemicity status. (TIF)

S1 Table Reported number of cases and cases per square kilometre. (DOCX)
