# Supplementary material for: Integrated morbidity mapping of lymphatic filariasis and podoconiosis cases in 20 co-endemic districts of Ethiopia
Source: PLoS Negl Trop Dis. 2018 Jul 2;12(7):e0006491. doi: 10.1371/journal.pntd.0006491 (PMC6044548; doi:10.1371/journal.pntd.0006491)
Supplement: S1 Checklist — (DOC) [file pntd.0006491.s001.doc]

STROBE Statement—Checklist of items that should be included in reports of ***cross-sectional studies***

|  | Item No | Recommendation |
| --- | --- | --- |
| **Title and abstract** | 1 | (*a*) Indicate the study’s design with a commonly used term in the title or the abstract  Abstract (paragraph 2 – methodology/principle findings) |
|  |  | (*b*) Provide in the abstract an informative and balanced summary of what was done and what was found  Abstract (paragraph 2 – methodology/principle findings) |
| Introduction | | |
| Background/rationale | 2 | Explain the scientific background and rationale for the investigation being reported  Introduction (paragraph 1, 2, 3, 4) |
| Objectives | 3 | State specific objectives, including any prespecified hypotheses  Introduction (paragraph 4 - line 100 – 103) |
| Methods | | |
| Study design | 4 | Present key elements of study design early in the paper  Methods section – ‘study design and tools’ |
| Setting | 5 | Describe the setting, locations, and relevant dates, including periods of recruitment, exposure, follow-up, and data collection  Methods section – ‘study site characteristics’ & ‘data collection and analysis’ |
| Participants | 6 | *(*a) Give the eligibility criteria, and the sources and methods of selection of participants  Methods section – ‘study design and tools’ & ‘data collection and analysis’ |
| Variables | 7 | Clearly define all outcomes, exposures, predictors, potential confounders, and effect modifiers. Give diagnostic criteria, if applicable  Methods section – ‘study design and tools’ |
| Data sources/ measurement | 8* | For each variable of interest, give sources of data and details of methods of assessment (measurement). Describe comparability of assessment methods if there is more than one group  Methods section – ‘study design and tools’ & ‘data collection and analysis’ |
| Bias | 9 | Describe any efforts to address potential sources of bias  Bias reduced by conducting training – methods section ‘training’  Bias reduced by having supervision – methods section ‘data collection and analysis’ |
| Study size | 10 | Explain how the study size was arrived at  Methods section - study site characteristics – paragraph 2, lines 131 to 134 |
| Quantitative variables | 11 | Explain how quantitative variables were handled in the analyses. If applicable, describe which groupings were chosen and why  Methods section – ‘data collection and analysis’ |
| Statistical methods | 12 | *(*a) Describe all statistical methods, including those used to control for confounding  Methods section – ‘data collection and analysis’ |
|  |  | (*b*) Describe any methods used to examine subgroups and interactions  Methods section – ‘data collection and analysis’ |
|  |  | (*c*) Explain how missing data were addressed  Missing data is taken note of in the results section i.e. results section – ‘acute attacks’ – line 275 -276 |
|  |  | (*d*) If applicable, describe analytical methods taking account of sampling strategy  N/A |
|  |  | (*e*) Describe any sensitivity analyses  N/A |
| Results | | |
| Participants | 13* | (a) Report numbers of individuals at each stage of study—eg numbers potentially eligible, examined for eligibility, confirmed eligible, included in the study, completing follow-up, and analysed  Results section – ‘summary of reported cases’ – also highlighted in Table 1 |
|  |  | (b) Give reasons for non-participation at each stage  N/A |
|  |  | (c) Consider use of a flow diagram  N/A |
| Descriptive data | 14* | (a) Give characteristics of study participants (eg demographic, clinical, social) and information on exposures and potential confounders  Results section – ‘summary of reported cases’ |
|  |  | (b) Indicate number of participants with missing data for each variable of interest  Missing data is taken note of in the results section i.e. results section – ‘acute attacks’ – line 275 -276 |
| Outcome data | 15* | Report numbers of outcome events or summary measures  Results section – ‘summary of reported cases’ |
| Main results | 16 | *(*a) Give unadjusted estimates and, if applicable, confounder-adjusted estimates and their precision (eg, 95% confidence interval). Make clear which confounders were adjusted for and why they were included  Provided within results section. ‘Severity of leg lymphoedema’ – line 260-261 |
|  |  | (*b*) Report category boundaries when continuous variables were categorized  Reported within tables i.e. age categories shown in Table 3 |
|  |  | (*c*) If relevant, consider translating estimates of relative risk into absolute risk for a meaningful time period  N/A |
| Other analyses | 17 | Report other analyses done—eg analyses of subgroups and interactions, and sensitivity analyses  Analyses by stratifying by age groups, sex, severity and acute attacks (Table 3 and Table 4) |
| Discussion | | |
| Key results | 18 | Summarise key results with reference to study objectives  Results section – paragraph 2 |
| Limitations | 19 | Discuss limitations of the study, taking into account sources of potential bias or imprecision. Discuss both direction and magnitude of any potential bias  Results section – paragraph 2 – lines 330 to 332 |
| Interpretation | 20 | Give a cautious overall interpretation of results considering objectives, limitations, multiplicity of analyses, results from similar studies, and other relevant evidence  Results section – paragraph 2, paragraph 4 |
| Generalisability | 21 | Discuss the generalisability (external validity) of the study results  Results section – paragraph 6, paragraph 7 |
| Other information | | |
| Funding | 22 | Give the source of funding and the role of the funders for the present study and, if applicable, for the original study on which the present article is based  Included in ‘funding information’ section on online submission |

*Give information separately for exposed and unexposed groups.

**Note:** An Explanation and Elaboration article discusses each checklist item and gives methodological background and published examples of transparent reporting. The STROBE checklist is best used in conjunction with this article (freely available on the Web sites of PLoS Medicine at http://www.plosmedicine.org/, Annals of Internal Medicine at http://www.annals.org/, and Epidemiology at http://www.epidem.com/). Information on the STROBE Initiative is available at www.strobe-statement.org.
