# Supplementary figures and images for: Integrated morbidity mapping of lymphatic filariasis and podoconiosis cases in 20 co-endemic districts of Ethiopia
Source: PLoS Negl Trop Dis. 2018 Jul 2;12(7):e0006491. doi: 10.1371/journal.pntd.0006491 (PMC6044548; doi:10.1371/journal.pntd.0006491)

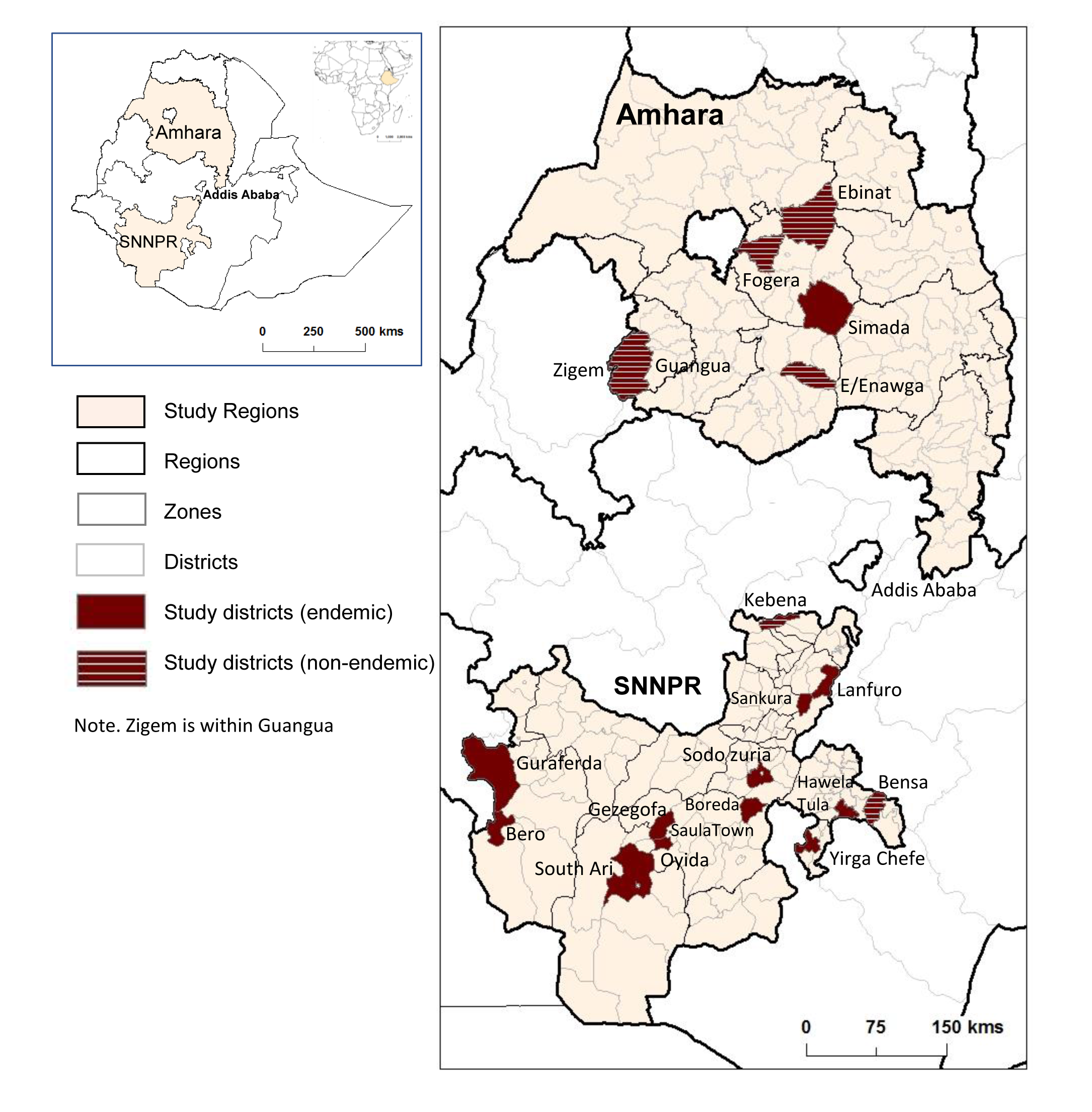

Supplement: S1 Fig — (TIF) [file pntd.0006491.s002.tif]
