# Supplementary material for: Integrated morbidity mapping of lymphatic filariasis and podoconiosis cases in 20 co-endemic districts of Ethiopia
Source: PLoS Negl Trop Dis. 2018 Jul 2;12(7):e0006491. doi: 10.1371/journal.pntd.0006491 (PMC6044548; doi:10.1371/journal.pntd.0006491)
Supplement: S1 Table — (DOCX) [file pntd.0006491.s003.docx]

S1 Table. Reported number of cases and cases per square kilometre.

| **Region** | **Zone** | **#** | **District** | **Area  (km²)** | **Leg lymphoedema only** | | **Hydrocoele only** | | **Both conditions*** | | **Breast lymphoedema only** | | **Total** | |
| --- | --- | --- | --- | --- | --- | --- | --- | --- | --- | --- | --- | --- | --- | --- |
|  |  |  |  |  |  |  |  |  |  |  |  |  |  |  |
|  |  |  |  |  | **N** | **Cases per km²** | **N** | **Cases per km²** | **N** | **Cases per km²** | **N** | **Cases per km²** | **N** | **Cases per km²** |
|  |  |  |  |  |  |  |  |  |  |  |  |  |  |  |
| SNNPR | Siliti | 1 | Lanfuro | 40.44 | 1,781 | 44.04 | 20 | 0.49 | 3 | 0.07 | 1 | 0.02 | 1,805 | 44.63 |
|  |  | 2 | Sankura | 21.76 | 1,130 | 51.94 | 23 | 1.06 | 17 | 0.78 | 0 | 0.00 | 1,170 | 53.78 |
|  | Sidama | 3 | Hawela Tula | 23.71 | 405 | 17.08 | 4 | 0.17 | 1 | 0.04 | 0 | 0.00 | 410 | 17.29 |
|  |  | 4 | Bensa ǂ | 42.21 | 1,882 | 44.58 | 54 | 1.28 | 29 | 0.69 | 2 | 0.05 | 1,967 | 46.60 |
|  | Gedeo | 5 | Yirga Chefe | 26.49 | 1,699 | 64.13 | 57 | 2.15 | 13 | 0.49 | 0 | 0.00 | 1,769 | 66.77 |
|  | Wollaita | 6 | Sodo zuria | 37.07 | 2,564 | 69.16 | 12 | 0.32 | 5 | 0.13 | 0 | 0.00 | 2,581 | 69.62 |
|  | GamoGofa | 7 | Boreda | 36.15 | 1,078 | 29.82 | 14 | 0.39 | 6 | 0.17 | 25 | 0.69 | 1,123 | 31.07 |
|  |  | 8 | Gezegofa | 39.45 | 1,343 | 34.04 | 11 | 0.28 | 32 | 0.81 | 2 | 0.05 | 1,388 | 35.18 |
|  |  | 9 | Oyida | 17.02 | 624 | 36.66 | 7 | 0.41 | 18 | 1.06 | 0 | 0.00 | 649 | 38.13 |
|  |  | 10 | Saula Town ǂ | 0.39 | 247 | 632.29 | 1 | 2.56 | 0 | 0.00 | 0 | 0.00 | 248 | 634.85 |
|  | Gurage | 11 | Kebena ǂ | 27.09 | 892 | 32.93 | 29 | 1.07 | 10 | 0.37 | 0 | 0.00 | 931 | 34.36 |
|  | Bench Maji | 12 | Bero | 48.19 | 105 | 2.18 | 0 | 0.00 | 0 | 0.00 | 0 | 0.00 | 105 | 2.18 |
|  |  | 13 | Guraferda | 202.59 | 663 | 3.27 | 12 | 0.06 | 4 | 0.02 | 0 | 0.00 | 679 | 3.35 |
|  | South Omo | 14 | South Ari | 169.97 | 2,277 | 13.40 | 41 | 0.24 | 101 | 0.59 | 42 | 0.25 | 2,461 | 14.48 |
| **SNNP regional total** | | | | **733** | **16,690** | **22.78** | **284** | **0.39** | **239** | **0.33** | **72** | **0.10** | **17,285** | **23.60** |
| Amhara | Awi | 15 | Zigem | 2.70 | 1,203 | 445.27 | 34 | 12.58 | 23 | 8.51 | 2 | 0.74 | 1,262 | 467.11 |
|  |  | 16 | Guanga ǂ | 192.71 | 1,487 | 7.72 | 38 | 0.20 | 54 | 0.28 | 3 | 0.02 | 1,582 | 8.21 |
|  | South Gondor | 17 | Ebinat ǂ | 208.69 | 1,080 | 5.18 | 112 | 0.54 | 6 | 0.03 | 0 | 0.00 | 1,198 | 5.74 |
|  |  | 18 | Fogera ǂ | 91.14 | 1,932 | 21.20 | 72 | 0.79 | 31 | 0.34 | 0 | 0.00 | 2,035 | 22.33 |
|  |  | 19 | Simada | 184.61 | 1,033 | 5.60 | 157 | 0.85 | 10 | 0.05 | 0 | 0.00 | 1,200 | 6.50 |
|  | East Gojam | 20 | E/Enawga ǂ | 78.12 | 1,483 | 18.98 | 53 | 0.68 | 24 | 0.31 | 0 | 0.00 | 1,560 | 19.97 |
| **Amhara regional total** | | | | **758** | **8,218** | **10.84** | **467** | **0.62** | **148** | **0.20** | **5** | **0.01** | **8,838** | **11.66** |
| **Overall total** | | | | **1,491** | **24,908** | **16.71** | **751** | **0.50** | **387** | **0.26** | **77** | **0.05** | **26,123** | **17.53** |
